# The Gingival Crevicular Fluid as a Source of Biomarkers to Enhance Efficiency of Orthodontic and Functional Treatment of Growing Patients

**DOI:** 10.1155/2017/3257235

**Published:** 2017-01-23

**Authors:** Mariana Caires Sobral de Aguiar, Giuseppe Perinetti, Jonas Capelli

**Affiliations:** ^1^Department of Orthodontics, Faculty of Dentistry, Rio de Janeiro State University, Rio de Janeiro, RJ, Brazil; ^2^Department of Medical, Surgical and Health Sciences, School of Dentistry, University of Trieste, Trieste, Italy

## Abstract

Gingival crevicular fluid (GCF) is a biological exudate and quantification of its constituents is a current method to identify specific biomarkers with reasonable sensitivity for several biological events. Studies are being performed to evaluate whether the GCF biomarkers in growing subjects reflect both the stages of individual skeletal maturation and the local tissue remodeling triggered by orthodontic force. Present evidence is still little regarding whether and which GCF biomarkers are correlated with the growth phase (mainly pubertal growth spurt), while huge investigations have been reported on several GCF biomarkers (for inflammation, tissue damage, bone deposition and resorption, and other biological processes) in relation to the orthodontic tooth movement. In spite of these investigations, the clinical applicability of the method is still limited with further data needed to reach a full diagnostic utility of specific GCF biomarkers in orthodontics. Future studies are warranted to elucidate the role of main GCF biomarkers and how they can be used to enhance functional treatment, optimize orthodontic force intensity, or prevent major tissue damage consequent to orthodontic treatment.

## 1. Introduction 

**Figure 1:**
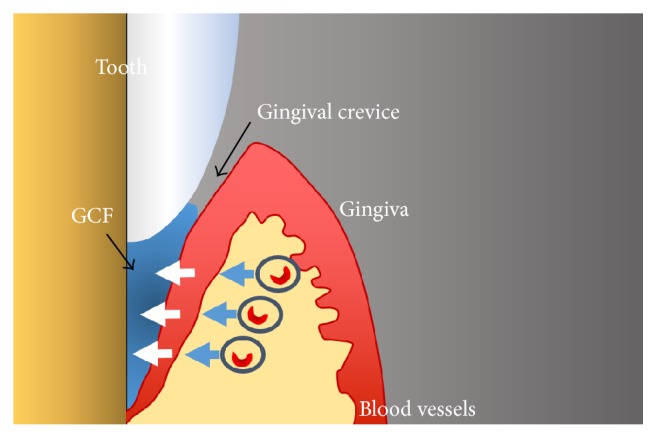
The gingival crevicular fluid (GCF) formation. The GCF flow is an interstitial fluid which appears in the crevice as a result of an osmotic gradient.

Gingival crevicular fluid (GCF) (Figure 1) is a biological exudate and quantification of its constituents is a current method to identify specific biomarkers with reasonable sensitivity [1]. Its formation was first defined by Alfano [2]. At sites in the absence of inflammation and subgingival plaque, the production of GCF is mediated by passive diffusion of the extracellular fluid by an osmotic gradient. In this situation, the GCF is considered as a transudate. When an inflammatory response is provoked by compounds of microbial origin, the permeability of the epithelial barrier and the underlying vasculature increases and the GCF protein concentration is now modulated by extent of plasma protein exudation. Subsequently, the GCF is considered an inflammatory exudate.

The GCF is a mixture of substances derived from serum, host inflammatory cells, structural cell of the periodontium, and oral bacteria [3, 4]. The molecules isolated from the sulcular fluid include electrolyte, small organic molecules, proteins, cytokines, specific antibodies, bacterial antigens, and enzymes of both host and bacterial origin [5, 6]. The host-derived substances in the GCF include antibodies, cytokines, enzymes, and tissue degradation products [7, 8].

The analysis of GCF is a very useful diagnostic instrument to both periodontology and orthodontics. The correlations between the levels of many host GCF biomarkers and periodontal diseases have been extensively studied and the predictive values for the biomarkers as summarized in Table 1 [9–12]. In orthodontics, biomarkers related to bone deposition (bone alkaline phosphatase and osteoprotegerin) represent new possibilities for the understanding of bone growth and remodeling [13]. The possibility of identifying the bone turnover in children and juvenile subjects can help orthodontists to decide when to intercept a malocclusion. The biomarkers found in GCF also permit the monitoring of the orthodontic movement and consequences of the forces applied through its level's variations.

The biomarkers found in GCF are interleukins, tumor necrosis factor-alfa prostaglandin E2, osteocalcin, RANK, OPG, RANKL, TGF-*β*1, matrix metalloproteinases, acid and alkaline phosphatase (ALP), aspartate aminotransferase (AST), IL-1RA, interferon-gamma, and others [14]. These biomarkers can be divided into six categories: biomarkers of cell death, tissue damage, inflammation, bone resorption, bone deposition and mineralization, and other biomarkers [14].

**Figure 2:**
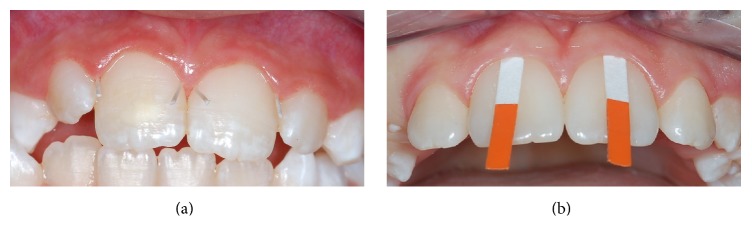
The gingival crevicular fluid collection with endodontic paper points (a) or periopapers (b).

There are three methods to collect the gingival crevicular fluid. The gingival washing technique consists of perfusing the GCF with an isotonic solution, as Hank's balanced solution, with fixed volume. The fluid collected represents a dilution of crevicular fluid, containing cells and soluble constituents, as plasma proteins. Another method is inserting capillary tubes, with specific diameter, into the entrance of the gingival crevice and the fluid migrates into the tube by capillary action [15]. However, the most used method for GCF collection is made with specifically designed absorbent filter paper as endodontic paper points or periopapers (Figure 2). The paper strips are inserted into the gingival crevice and left in situ for 5 to 60 seconds to allow the GCF to be adsorbed by the paper [16].

The purpose of this review is to identify the biochemical markers present in the gingival crevicular fluid and their relevance to identify the growth phase and as well analyze the expression of the biomarkers during the orthodontic movement in children and young subjects.

## 2. Identification of Growth Phase

The decision to intercept orthopedically on a growing patient depends primarily on the identification of his skeletal maturation phase. The most desirable time for treatment is different in various malocclusions [17–19].

Different established methods are used to identify the growth phase. The analysis of cervical vertebra maturation (CVM) is a method based on assessing the shape of the cervical bodies, as seen in lateral cephalograms. The CVM method shows great reliability, according to Baccetti et al. [20], Franchi et al. [21], and Rainey et al. [22]. Another radiographic method is the hand-wrist analysis that calculates the mean age for the appearance of each of the various centers of ossification or the epiphyseal closure and variations in these ages [23, 24].

Alternative methods to identify the growth phase are analysis of dentition [25], chronological age [26, 27], and dental maturation [28, 29]. These methods are mainly morphological and recent studies affirm that those are not reliable assessments of growth phases [30, 31]. New possibilities might be offered by the biochemical markers. Collection of gingival crevicular fluid avoids radiographic exposure and the biomarkers represent agents that are directly involved in bone growth and remodeling [1].

The alkaline phosphatase (ALP) has been investigated as reliable biologic indicator of skeletal maturation in different studies, where the ALP levels are compared with other methods to identify the skeletal maturation in growing patients [32, 33]. The bone alkaline phosphatase is synthesized by the osteoblasts and is presumed to be involved in the calcification of bone matrix. It is considered to be a highly specific marker of the bone-forming activity of osteoblasts.

Perinetti et al. [33] compared the relation between the cervical vertebra maturation and the level of ALP in the gingival crevicular fluid in patients with age range 7.8–17.7 years. The enzyme activity greater level was detected in CS3 and CS4 phases that are correspondent to the peak in the mandibular growth in the CVM analysis. As reported by Szulc et al. [34], serum ALP activity, which is the most used biochemical marker for bone turnover, increases at puberty and decreases in adulthood. Neither dentition phase or chronological age show significant correlations with the skeletal maturation phases, as monitored through the GCF ALP activity, according to Perinetti and contardo [35]. The authors have concluded that the treatment of dentofacial disharmonies in individual patients should not rely on the clinical parameters of dentition and chronological age. This conclusion is the opposite of a recent Indian study, which affirms that there is a positive correlation between chronological age and cervical vertebrae skeletal maturation [36].

## 3. Monitoring of Orthodontic Tooth Movement

The orthodontic tooth movement is possible by the application of a controlled mechanical force and it results in biologic reactions that alter the surrounding dental and periodontal tissues [37]. These alterations include a cascade of events—in the mineralized (alveolar bone) and nonmineralized (periodontium) tissues—that allow the tooth movement. Biochemical markers representing these biological modifications are expressed during specific phenomena, that is, simile-inflammatory process, bone resorption and formation, periodontal ligament changes, and vascular and neural responses [38].

Monitoring the levels of biochemical markers during orthodontic movement might be a useful procedure for clinicians to analyze the degree of bone remodeling. Gingival crevicular fluid reflects the immune reactions, interactions host-parasite [39], and reactions to biochemical stress [40].

Interleukins are particularly important for consequent tooth movement, because these cytokines stimulate osteoclast formation and bone resorption promoted by preformed osteoclasts. Interleukins can be classified as proinflammatory or anti-inflammatory. Proinflammatory interleukins are interleukin-1 *β* (IL-1*β*), interleukin-2, interleukin-6, and interleukin-8 and the anti-inflammatory are interleukin-1, receptor antagonist (IL-1RA), interleukin-4, interleukin-10, and interleukin-13. Interleukin-1 *β* (IL-1 *β*) is a cytokine that stimulates the bone resorption [41] and its concentration 24 hours after the beginning of tooth movement increases, according to Uematsu et al. [42]. These authors also demonstrated an increase in the levels of other proinflammatory cytokines, as IL-6 and tumor necrosis factor-alpha. IL-6 is a cytokine originated from macrophages and T-cells. When children and adult subjects, undergoing orthodontic treatment, had their GCF compared, children showed a higher mean concentration of IL-6 than the adults 24 hours after the beginning of the movement [42].

Tooth movement also requires the binding of receptor activator of nuclear factor kappa *β* ligand (RANKL) to receptor activator of nuclear factor kappa (RANK), a cell membrane protein found on osteoclast precursor cells [43]. RANK is a cell membrane protein found on osteoclast precursor cells while the RANKL is a protein produced by the osteoblasts. During the orthodontic movement, RANKL is responsible for the generation and maintenance of osteoclasts by binding RANK [3]. On the other hand, osteoprotegerin (OPG) acts as a decoy receptor that binds to RANKL and blocks osteoclastogenesis [43].

The RANK-RANKL-OPG system is of primary importance to osteoclast differentiation during orthodontic movement. The levels of RANKL in GCF during the movement have increased, while the levels of OPG decreased, especially in the first 24 hours after the application of orthodontic force, which suggests bone resorption [43, 44]. A study compared the effects of aging on RANKL and OPG levels in gingival crevicular fluid during orthodontic tooth movement and as a result, it was found that juvenile patients had a higher amount of tooth movement when compared with that of adults, after 168 h of the beginning of treatment. That difference could be related to a lower RANKL/OPG ratio in GCF in adult patients during the early stages of orthodontic movement and suggests that is the reason the movement is faster in young patients [44].

When an orthodontic force is applied to a tooth, it creates areas of tension and compression in the periodontal ligament (PDL). The mechanical stress changes the vascularity and blood flow within the PDL, which allow the remodeling of the PDL.

Bone-forming cells have been shown to have alkaline phosphatase (ALP) activity and changes in this enzyme in serum and bone have been used as markers for bone metabolism in several diseases [45, 46]. During orthodontic treatment, acid and alkaline phosphatase in human GCF have been correlated with the total appliance duration. GCF ALP has a primary role in bone mineralization, because it hydrolyses inorganic pyrophosphate, which is an inhibitor of the mineralization process. The ALP has been shown to be sensitive to alveolar bone formation during orthodontic tooth movement [47, 48]. A split-mouth prospective study [49] in prepubertal subjects was made to monitor alveolar bone formation at the tension sites of the first molars undergoing rapid maxillary expansion (RME) treatment. In this study, the GCF ALP activity was used as a biomarker of tissue remodeling to determine the existence and duration of active alveolar bone formation during the retention phase. The authors have concluded that during the retention phase of RME, there is an increase in GCF ALP activity in the tensions sites, at both 3 and 6 months.

Perinetti et al. [48] investigated the ALP activity in GCF and analyzed if this enzyme can be a diagnostic method to assess the orthodontic movement. In this split-mouth study, the maxillary first molars under treatment served as a test in each patient, with one being retracted, and the contralateral molars were not subjected to distal forces. Thus, they showed that GCF ALP activity was greater in the distalized molars than that in the nonmoved contralateral molars. The ratio of the activity of the ALP was higher in tension sites, when compared with the compression sites. As a conclusion, they suggested that the ALP activity in GCF reflects the biologic activity in the periodontium during orthodontic movement. The GCF ALP was tested in other studies [50, 51], which also showed a higher level of ALP in the tension sites, after application of orthodontic force, and confirmed this enzyme as a biomarker of orthodontic movement.

Prostaglandin E2 (PGE2) is produced by the periodontal ligament cells and it is a proinflammatory mediator. PGE2 acts as biochemical mediator of bone resorption induced by the orthodontic movement, stimulating the osteoclastic activity. This biomarker is known to be a potent stimulator of bone resorption and its production is controlled in part by IL-1 [3]. Grieve III et al. [52] showed that PGE2 and IL-1*β* were significantly elevated after the initial tooth movement but returned to baseline levels after seven days. Ren et al. [7] showed that the concentrations were significantly elevated after 24 hours of activation in juvenile and adult patients but concluded that the mediator levels in juvenile subjects are more responsive than the levels in adults. In agreement with this conclusion, another study [53] showed that the levels of PGE2 were higher in young subjects than in the older patient group. This could be an explanation of why the speed of orthodontic treatment may be different in adults versus juveniles. The hypothesis is that, in juveniles, the inflammatory responses can react faster to local changes.

Tumor necrosis factor-*α* (TNF-*α*) is a proinflammatory cytokine that can be derived from both monocyte and macrophage. TNF-*α* stimulates proteolytic enzyme synthesis and osteoclastic activity, so it is involved in bone resorption. It is also an apoptotic factor for osteocytes, which could be the signal for osteoclast recruitment to resorb bone in the side undergoing PDL pressure, while it simultaneously inhibits osteoblasts [3]. TNF-*α* also controls the appearance of osteoclasts at compression sites. In a study [54] with juveniles patients (16–19 years old) who need orthodontic treatment with molar distalization, the levels of TNF-*α* and IL-1*β* were assessed and there were increases in their concentrations and also an increase in GCF volume. Lowney et al. [55] also studied the expression of TNF-*α* and found an increase in its expression during orthodontic treatment.

The major noncollagenous components of bone in serum and a product of the osteoblast activity, osteocalcin, have been used as a marker of bone formation, but there are several factors that complicate interpretation of the results. Nevertheless, assays for intact osteocalcin have been shown to be related to growth velocity in children [56]. The ALP and osteocalcin levels were also investigated in a group of girls during puberty, with ages between 11.6 and 15.5 years and it showed that the increase in levels of bone specific alkaline phosphatase, osteocalcin, and urinary deoxypyridinoline suggests that these markers may be relatively more sensitive as indicators of skeletal health during puberty [57].

Osteoprotegerin (OPG) is a member of the tumor necrosis factor receptor family and a soluble decoy receptor against RANKL. It is produced by osteoblasts and other cells and a key factor in the inhibition of osteoclast differentiation and activation [58]. Nishijima et al. [59] analyzed the levels of RANKL and OPG in GCF during orthodontic movement in adolescent patients. They showed that RANKL levels increased during the treatment and in contrast, the OPG levels decreased. The changes in these cytokines may be involved in bone resorption as a response to compression force.

The soft tissue is also remodeled following orthodontic tooth movement. These tissues are metabolized by various enzymes, including matrix metalloproteinases (MMPs) and tissue inhibitors of matrix metalloproteinases (TIMPs). Collagenases, MMP-1 and MMP-8, degrade collagen fibers, whereas gelatinases (MMP-2 and MMP-9) degrade denatured collagen, complementing collagenases [60]. In humans, GCF MMP-1 and MMP-8, MMP-2, and MMP-9 [61] and TIMP-1 [62] have all been shown to increase at sites of compression and tension. Therefore, crevicular MMP-9 may also serve as biomarker to monitor remodeling of the periodontal tissues during tooth movement [63].

## 4. Concluding Remarks

The GCF is a well-known source of biomarkers with potential applications in both periodontology and orthodontics. Its analysis permits the orthodontists to identify the consequences of orthodontic forces in paradental tissues (periodontal ligament and alveolar bone). The GCF biomarkers may be also helpful to assess the growth phase in children and juvenile patients. Main advantages of this method are that it can be done in private dentist offices, is quick, and avoids radiographic exposure. However, despite all the reported investigations, the clinical applicability of the method is still limited with further data needed to reach a full diagnostic utility of specific GCF biomarkers for orthodontics. Therefore, more studies are warranted to elucidate the role of main GCF biomarkers and how the quantification of which may be used to enhance functional treatment, optimize orthodontic force intensity, or prevent major tissue damage consequent to orthodontic treatment. In this view, biochemical monitoring related to orthodontic treatment represents a promising issue.

## Figures and Tables

**Table 1:** Main GCF host biomarkers according to biological significance.

Bone deposition and mineralization
Bone alkaline phosphatase
Osteoprotegerin
Bone resorption
Osteonectin
Bone phosphoprotein
Osteocalcin
Cross-linked carboxyterminal telopeptide of type I collagen
Receptor activator of nuclear factor kappa-B and its ligand
Inflammation
Cytokines (interleukins, tumor necrosis factors, interferons, growth factors, and colony-stimulating factors)
Arachidonic acid derivates (prostaglandins, leukotrienes)
Neutrophil alkaline phosphatase
Hydroxyproline
Collagen cross-linking peptides
Others
Cell death or tissue damage
Aspartate aminotransferase
Lactate dehydrogenase
Hydroxyproline
Collagen cross-linking peptides
Glycosaminoglycans
Metalloproteases (proteolytic enzyme)
Cathepsin B (proteolytic enzyme)
Antibodies
